# *Methylobacterium*-Induced Endophyte Community Changes Correspond with Protection of Plants against Pathogen Attack

**DOI:** 10.1371/journal.pone.0046802

**Published:** 2012-10-03

**Authors:** Pavlo Ardanov, Angela Sessitsch, Hely Häggman, Natalia Kozyrovska, Anna Maria Pirttilä

**Affiliations:** 1 Department of Biology, University of Oulu, Oulu, Finland; 2 Bioresources Unit, AIT Austrian Institute of Technology GmbH, Tulln, Austria; 3 Institute of Molecular Biology and Genetics of NASU, Kyiv, Ukraine; University of California Merced, United States of America

## Abstract

Plant inoculation with endophytic bacteria that normally live inside the plant without harming the host is a highly promising approach for biological disease control. The mechanism of resistance induction by beneficial bacteria is poorly understood, because pathways are only partly known and systemic responses are typically not seen. The innate endophytic community structures change in response to external factors such as inoculation, and bacterial endophytes can exhibit direct or indirect antagonism towards pathogens. Earlier we showed that resistance induction by an endophytic *Methylobacterium* sp. in potato towards *Pectobacterium atrosepticum* was dependent on the density of the inoculum, whereas the bacterium itself had no antagonistic activity. To elucidate the role of innate endophyte communities in plant responses, we studied community changes in both *in vitro* and greenhouse experiments using various combinations of plants, endophyte inoculants, and pathogens. Induction of resistance was studied in several potato (*Solanum tuberosum* L.) cultivars by *Methylobacterium* sp. IMBG290 against the pathogens *P. atrosepticum*, *Phytophthora infestans* and *Pseudomonas syringae* pv. tomato DC3000, and in pine (*Pinus sylvestris* L.) by *M. extorquens* DSM13060 against *Gremmeniella abietina.* The capacities of the inoculated endophytic *Methylobacterium* spp. strains to induce resistance were dependent on the plant cultivar, pathogen, and on the density of *Methylobacterium* spp. inoculum. Composition of the endophyte community changed in response to inoculation in shoot tissues and correlated with resistance or susceptibility to the disease. Our results demonstrate that endophytic *Methylobacterium* spp. strains have varying effects on plant disease resistance, which can be modulated through the endophyte community of the host.

## Introduction

Plant inoculation with nonpathogenic bacteria may induce faster defense reactions towards bacterial, fungal, or viral pathogens and environmental stresses [1], [2]. The most studied resistance-inducing bacteria are soil-dwelling rhizobacteria, which usually do not enter plant tissues, but live in close proximity to, or as epiphytes on the plant root surface. These bacteria may induce systemic resistance (ISR) via jasmonic acid (JA) and/or ethylene (ET) signaling pathways of the plant [1], [3]. Overall, the molecular mechanisms behind induction of resistance by rhizobacteria remain unclear. There are no consistent changes in the expression of plant genes encoding pathogenesis-related proteins, which are activated in the systemic acquired resistance (SAR) triggered by necrotizing pathogens [1], [3]. Because systemic changes are not detected in the plant expression profile before challenge inoculation with the pathogen, the plant responses have been characterized as “priming” of the plant defense system [1], [3].

Some rhizobacteria can also enter and colonize the root and above-ground tissues internally as endophytes. Endophytic bacteria are generally defined to colonize the plant interior without harming the host [4], and a number of endophytes are able to establish a mutualistic relationship with the host by promoting plant growth [2], [5], [6]. Each plant individual contains communities of endophytic populations in each tissue, and for example the root and shoot communities typically differ from each other [4], [6]–[9]. Some members of these innate (or resident) endophyte communities are transmitted through the seeds or vegetative parts from parent to progeny, and others enter the plant during the life cycle [4], [6].

Results of several studies suggest that innate endophytic communities can be directly involved in plant defense [5], [10]–[13]. Endophytes can reduce pathogen invasion by various mechanisms, from outcompeting phytopathogens to the production of a wide range of compounds against the invader, and by induction of plant resistance. Numerous reports indicate that endophytes have direct antagonistic potency towards other microbes. This trait is essential for defense of the endophyte itself and can increase plant defense, depending on the species and cultivar [5], [10], [14], [15]. Endophytic bacteria also have the potential to complement the inefficient antioxidative systems of some plant species with their own reactive oxygen species (ROS) -eliminating mechanisms, and/or activate the antioxidant system of the plant [16]–[18]. The outcome of the concerted action of the plant and the endophytes depends on the structure of the endophyte population.

The structure of innate endophytic bacterial community depends on the genotype [19] and the developmental stage of the plant [20]–[22], and is shaped by infection by pathogens [10], [14], [23], beneficial bacteria [5], [11], [21], [22], [24]–[26], or by other environmental factors [4], [8], [23], [27]. In our earlier studies, inoculation of *in vitro*-grown potato with the rhizosphere strain *Pseudomonas fluorescens* IMBG163 resulted in outgrowth of endophytic *Methylobacterium* sp. (later named strain IMBG290) from the plant tissue [11]. This suggests that the interaction between microbes can have drastic effects on the plant.

*Methylobacterium* spp. are frequently encountered as endophytes and have the capacity for methylotrophy, biofilm formation, production of quorum-sensing signals, heavy metal and other stress resistance, and ISR [11], [18], [28]–[30]. Recently, we found that *Methylobacterium* sp. IMBG290 can induce resistance in potato towards *Pectobacterium atrosepticum* by activation of the antioxidant system in an inoculum density-dependent manner [17]. Resistance was induced when a low density of endophytes was used for inoculation, whereas high density resulted in susceptibility to the pathogen. The inoculation with *Methylobacterium* sp. IMBG290 itself caused no symptoms of disease or defense reaction [17], but inoculation of endophytic *M. extorquens* DSM13060 has been shown to induce the expression of plant defense genes in pine [31]. The interaction between the plant, the inoculated endophyte and the environment could be very complex and might further affect the existing endophyte communities. The aim of this study was to investigate how the inoculation of *Methylobacterium* spp. endophytes to potato (*Solanum tuberosum* L) and pine (*Pinus sylvestris* L.) affects plant responses to different phytopathogens, and how these interactions shape the existing endophytic microflora. The endophyte community changes were analyzed by terminal restriction fragment length polymorphism (T-RFLP) - based community fingerprinting, where changes in the relative abundance of community members were detected as corresponding terminal fragments (T-RF) [32], [33].

## Materials and Methods

### Experimental Design

The disease resistance inducing capacity of two *Methylobacterium* spp. strains, IMBG290 and DSM13060, was studied on their native hosts potato and pine in *in vitro* (both species; experiment 1) and greenhouse conditions (potato; experiment 2). The effect of *Methylobacterium* spp. inoculation on the innate endophytic bacterial community of the plant host was studied by T-RFLP and compared with the effect of pathogen infection. Additionally, the genotype-dependent changes were assessed in potato. Summary of the experiments is shown in Table S1. All experiments were performed with five biological replicates (*n*). Each replicate represents all plants grown in one pot, *e.g.* five plants (potato *in vitro* experiment), seven plants (potato greenhouse experiment) and fifteen plants (pine *in vitro* experiment).

### Plant Material and Culture Conditions

Potato plants (*Solanum tuberosum* L. cvs. Blue Congo, Timo, Pito, Matilda; The Seed Potato Centre, Tyrnävä, Finland) were propagated for the *in vitro* experiment by micrografting and cultivated on Murashige and Skoog (MS) [34] agar medium without phytohormones in the growth chamber, where the growth conditions were 16/8 hours light/dark photoperiod (34–40×µmol/m^2^s), 22°C and relative humidity of 80–85%. For the greenhouse experiment, micrografts of *in vitro* potato cvs. Bellarosa and Javir (Institute for Potato Research, Nemishaeve, Ukraine) were grown on perlite saturated with MS salts solution in the growth chamber before transfer to greenhouse.

For Scots pine (*Pinus sylvestris* L.), open-pollinated seed material (EY/FIN/M24-97-0203) was obtained from Siemen Forelia Oy, Rovaniemi, Finland. To remove epiphytic microbes, seeds were kept at 55°C for 3 days, soaked in sterile water for 12 h, then sterilized with 4% calcium hypochlorite (Sigma-Aldrich, USA) for 12 min and rinsed with sterile water three times. The seeds were cultivated in the growth chamber on sterile vermiculite saturated with deionized water.

### Microbial Strains and Culture Conditions

*Pseudomonas syringae* pv. tomato DC3000, *Pectobacterium atrosepticum, Methylobacterium* sp. IMBG290 and *Methylobacterium extorquens* DSM13060 were cultured as described previously [17]. *Phytophthora infestans* was cultivated according to the Eucablight protocol (http://www.eucablight.org/EucaBlight.asp) and *Gremmeniella abietina* according to Petäistö & Kurkela [35]. In general, selective media were used for microbial cultivation and methanol was used as the carbon source for the *Methylobacterium* spp. strains. *P. infestans* was cultured on detached potato leaves to induce sporulation.

### Plant Inoculation with Endophytic *Methylobacterium* spp. Strains

The plants were inoculated with endophyte strains originally derived from the same plant species, *Methylobacterium* sp. IMBG290 for potato and *M. extorquens* DSM13060 for pine, by standard procedures found most efficient for each species. For the *in vitro* experiment, potato cuttings were treated with a suspension of *Methylobacterium* sp. IMBG290 in 10 mM MgSO_4_ (10^5^, 10^6^, 10^7^ or 10^8^ CFU mL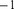) for 20 min. For the greenhouse experiment, *Methylobacterium* sp. IMBG290 cells (10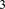 and 10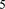 CFU g
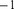
in individual treatments) were re-suspended in MS salts solution and mixed with perlite (grain size: 1.5–5 mm). For pine, two-month old seedlings were watered with a suspension of *M. extorquens* in 10 mM MgSO_4_ (10^4^, 10^5^, 10^6^, 10^7^ or 10^8^ CFU mL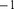). Controls were mock-treated with 10 mM MgSO_4_. Lower inoculum densities were used for the greenhouse experiment because *Methylobacterium* spp. cells were inoculated directly to the nutrient-rich substrate, assuming better bacterial growth in the ambient conditions and continuous plant colonization from the substrate.

### Colonization Assay

To study *Methylobacterium* spp. colonization by a culturing assay, a kanamycin-resistant strain of *Methylobacterium* sp. IMBG290, produced by transposon mutagenesis, was used for treatment of potato cv. Blue Congo plants, as described above. After four weeks, whole plants were surface sterilized with 70% ethanol and 5% sodium hypochlorite and flamed [32] prior to maceration in 10 mM MgSO_4_. Serial dilutions were plated on a selective medium [17] with kanamycin, and pink-pigmented colonies were counted.

### Pathogen Inoculation

A challenge inoculation with the pathogen was performed four weeks after endophyte inoculation. Inoculation with the pathogens *P. syringae* pv. tomato and *P. atrosepticum* was performed as described earlier [17]. In the case of the greenhouse plants, the pathogen suspension was sprayed. The foliage blight test and the detached leaf test of *P. infestans* were performed on *in vitro-*grown and greenhouse plants, respectively, according to the Eucablight protocol (http://www.eucablight.org/Protocol/Protocol.asp). With respect to *G. abietina* infection, pine shoots were soaked in a suspension of fungal conidia (10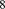 conidia mL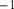) in 10 mM MgSO_4_ with 0.025% (v/v) Silwet L-77. The typical disease symptoms (necrotic spots on potato leaves, chlorotic and necrotic leaf symptoms, sporulating lesions with leaf necrosis, and needle necrosis for *P. atrosepticum*, *P. syringae* pv. tomato, *P. infestans* and *G. abietina*, respectively) were recorded five days (except nine days for *G. abietina*) after pathogen infection. The percentage of symptomatic leaves per plant (or the percentage of symptomatic seedlings per pot on *G. abietina*) was calculated.

### DNA Extraction and T-RFLP

Endophytic microbial communities were analyzed from plants four weeks after endophyte inoculation (prior to pathogen application) and three days after pathogen inoculation. Whole *in vitro*-grown plants and 5–7 cm long shoot tips of greenhouse-grown plants were surface sterilized as described for the colonization assay. In experiment 1, the plant material was used for a bead-beating step prior to DNA isolation [32]. In experiment 2, the plant material was macerated in a mortar with glass beads because a bead mill was not available, and the liquid extract was used for the DNA isolation. The DNA was extracted by the CTAB method [36]. For pine, the extraction buffer was supplemented with 2% polyvinylpolypyrrolidone (Serva, Germany) (w/v) and 2% 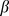-mercaptoethanol (Sigma-Aldrich, USA) (v/v).

Bacteria-specific primers, as well as restriction enzymes, were tested for their suitability for the T-RFLP analysis using the MICA software [37]. Bacterial 16S rRNA gene was amplified by PCR using the primers 799f [9] and 1520r [38], which generate different sizes of bacterial and mitochondria-derived amplicons, whereas chloroplast and other plant-derived rRNA genes are not amplified. For experiment 1, the forward primer was 5′-labeled with 6FAM™, while the reverse primer was unlabeled. For experiment 2, both primers were labeled at 5′ end to increase resolving power of the method, the forward primer with NED™ and the reverse primer with VIC® (primers were purchased from Applied Biosystems, Espoo, Finland). The DNA amplification, extraction of the PCR product from the gel, restriction digestion and processing for T-RFLP analysis were performed according to Sessitsch & Rasche [32]. PCR products from three individual reactions from each sample were pooled together and 50 ng of the pooled PCR product was digested with *Alu*I and *Hha*I (Fermentas, Lithuania) for experiments 1 and 2, respectively. The fragments were separated on an ABI3100 sequencer using POP4 polymer and an internal size standard GS500 ROX (Applied Biosystems, UK) in experiment 1 and ABI3130 sequencer, POP6 polymer and GS600 LYZ size standard in experiment 2 (depending on which instrument was in use at each research site).

### Sequence Analysis

The 16S rRNA genes were amplified as described above with unlabeled primers. Cloning and sequencing were performed as described by Sessitsch & Rasche [32]. The T-RFLP results indicated a low number of bacterial species in *in vitro*-grown plants, as there were only small differences between T-RFs of the cultivars and treatments, and therefore several *in vitro-*grown potato and pine samples were pooled together and used for DNA amplification and sequencing. To avoid sequencing of identical clones, the clones (120 for potato and 16 for pine) were first analyzed by amplified ribosomal DNA restriction analysis (ARDRA) using *Alu*I as the restriction enzyme. Unique bands were sequenced using Big Dye Terminator v3.1 Cycle Sequencing kit (Applied Biosystems, Foster City, CA, USA). Extension products were then purified by the ethanol/EDTA precipitation protocol and analyzed on a ABI 3100 Avant Genetic analyzer (Applied Biosystems, Foster City, CA, USA) as recommended by the manufacturer. Chimera check was done with Pintail software v.1.1 (Cardiff School of Biosciences, UK). Sequences were subjected to Basic Local Alignment Search Tool (BLAST) analysis with the National Center for Biotechnology Information (NCBI, Bethesda, MD) database.

### Nucleotide Accession Numbers

The bacterial sequences were deposited in the NCBI database under accession numbers **GU939191**–**GU939194, JN897354.**

### Data Processing and Statistical Analyses

The presented data of biotests are mean values 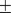
SD. The statistical significance of the differences between mean values was determined by the Student’s *t*-test. T-RFLP data were collected using the Peak Scanner Software v1.0 (Applied Biosystems, UK). The peaks were determined at 40–450 bp using Local Southern methods for size calling [39] and the baseline was set to 5 fluorescent units. Each fluorogram was additionally checked visually to ensure proper peak capture, as well as discreteness of the closely sized peaks. Further statistical analysis was performed using the T-REX software [40]. True peaks were determined according to Abdo *et al.* [33]. The data were normalized and the data matrix was constructed using peak height averaged over replicates (Table S2). T-RFs occurring in less than 5% of the samples were omitted. ANOVA was performed on the data matrix, and the beta diversity and the percentages of the main and interaction effects were determined. The data were analyzed by Additive Main Effects and Multiplicative Interaction Model [40].

## Results

Capacity of the *Methylobacterium* spp. endophytes to induce resistance was tested in different conditions towards different pathogens (Table S1). The treatments resulting in specific and profound profiles of *Methylobacterium* -triggered resistance were selected to study the endophytic bacterial communities by T-RFLP.

### Potato (Solanum tuberosum L.)

#### *In vitro* experiment

**Figure 1:**
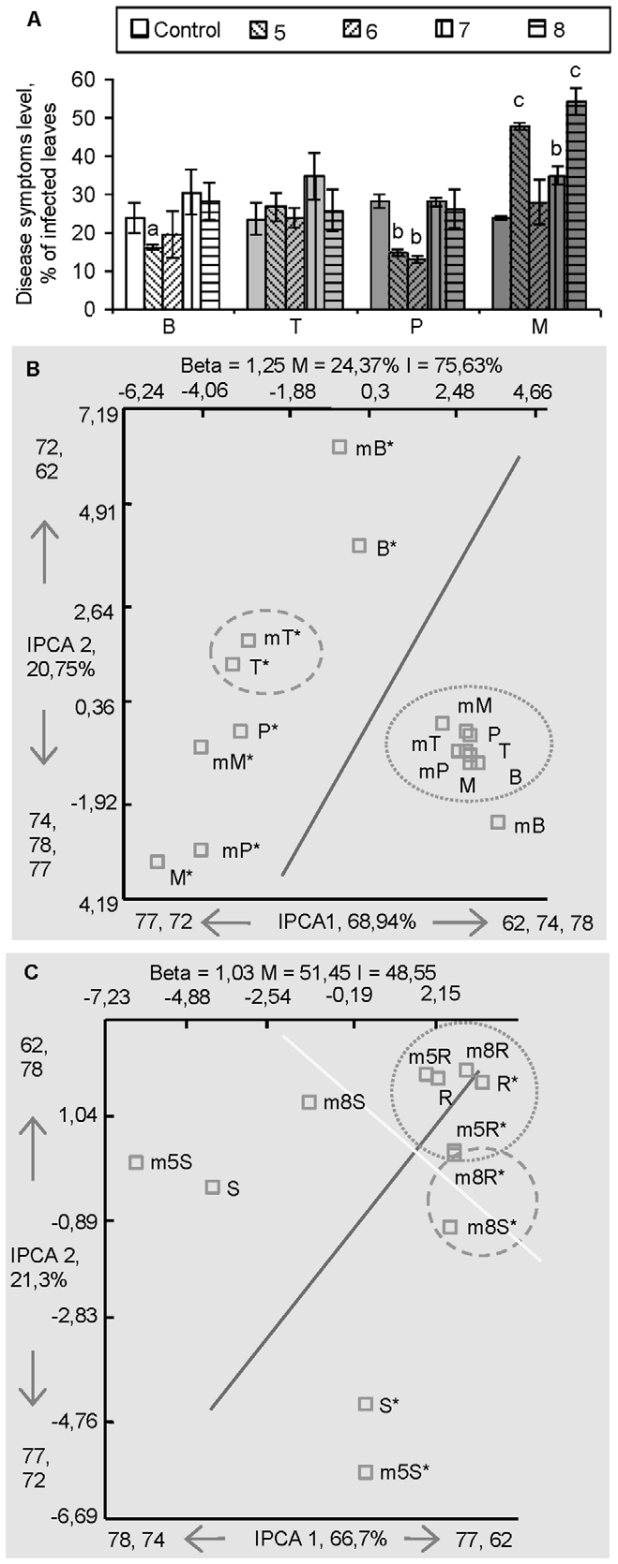
Disease resistance and terminal fragment length polymorphism (T-RFLP) analysis of endophyte communities in *Methylobacterium*-inoculated *in vitro-*grown potato plants. (A) Resistance of *in vitro-*grown potato cvs. Blue Congo, Timo, Pito, Matilda (B, T, P, M) to *Pectobacterium atrosepticum* induced by *Methylobacterium* sp. IMBG290 applied at densities 10^5^ 10^6^, 10^7^ and 10^8^ CFU ml^−1^ (5, 6, 7 and 8). (B) T-RFLP analysis of bacterial communities of shoots of the different potato cultivars at 10^5^ inoculation density of *Methylobacterium* sp. IMBG290 (m) where asterisk indicates challenge inoculated plants. (C) T-RFLP analysis of bacterial communities of shoots (S) and roots (R) of Blue Congo inoculated at 10^5^ and 10^8^ densities. Disease resistance data are mean ± SD (*n* = 5), letters indicate significant difference between treatments and control by Student’s *t*-test (a, b and c indicate P<0.05, 0.01 and 0.001, respectively). Cluster plots generated by Additive Main Effects and Multiplicative Interaction (AMMI) analysis are constructed from three T-RFLP replicates and contain the information on beta diversity (Beta), the percentage of the main (M) and interaction (I) effects, the principal T-RFs responsible for the data ordination for each of the interaction principal components axes (IPCA1 and 2), and the percentage of variance captured by each of the axes. Different shapes indicate grouping patterns.

The *Methylobacterium* sp. IMBG290 was tested in potato cultivars Blue Congo, Timo, Pito and Matilda *in vitro* against two pathogens, the bacterial soft-rot pathogen *P. atrosepticum* and the oomycete *P. infestans*. Against *P. infestans* only Pito cultivar exhibited some resistance (Fig. S1A), but a range of resistance levels was observed towards *P. atrosepticum* (Fig. 1A) depending on the cultivar and on the inoculation density of *Methylobacterium* sp. IMBG290. Enhanced resistance was found in the cultivars Blue Congo and Pito when *Methylobacterium* sp. IMBG290 was applied at low densities (10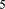 to 10^6^ CFU ml^−1^), but not in the cultivar Timo. Enhanced susceptibility to the pathogen was observed in the cultivar Matilda (Fig. 1A). These experiments were selected for T-RFLP analysis of the innate endophyte communities before and after endophyte and challenge inoculations. The comparison of shoot endophyte communities was made between potato genotypes treated with 10^5^-inoculation density of *Methylobacterium* sp. IMBG290 (Fig. 1B), and shoot and root communities were compared in Blue Congo cultivar treated with low (10^5^) and high (10^8^) inoculation densities (Fig. 1C).

**Figure 2:**
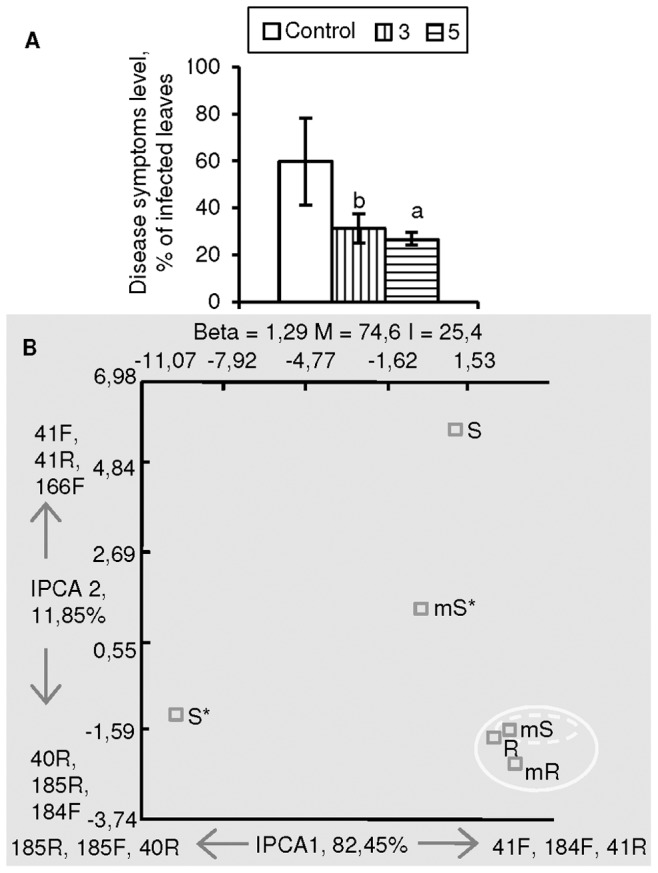
Disease resistance and terminal fragment length polymorphism (T-RFLP) analysis of endophyte communities in *Methylobacterium*-inoculated greenhouse-grown potato plants. (A) Resistance of greenhouse-grown potato cv. Bellarosa to *Pseudomonas syringae pv.* tomato DC3000 induced by *Methylobacterium* sp. IMBG290 at densities 10^3^ (3) and 10^5^ (5) CFU ml^−1^ and (B) analysis of the corresponding bacterial communities (combined data of labeled forward (F) and reverse (R) T-RFs of the amplicon) of shoots and roots at inoculation density of 10^5^. Disease resistance data are means ± SD (*n* = 5), letters indicate significant difference between treatments and control by Student’s *t*-test (a, b and c indicate P<0.05, 0.01 and 0.001, respectively). Cluster plots generated by Additive Main Effects and Multiplicative Interaction (AMMI) analysis are constructed from three T-RFLP replicates and contain the information on beta diversity (Beta), the percentage of the main (M) and interaction (I) effects, the principal T-RFs responsible for the data ordination for each of the interaction principal components axes (IPCA1 and 2), and the percentage of variance captured by each of the axes. Different shapes indicate grouping patterns.

**Figure 3:**
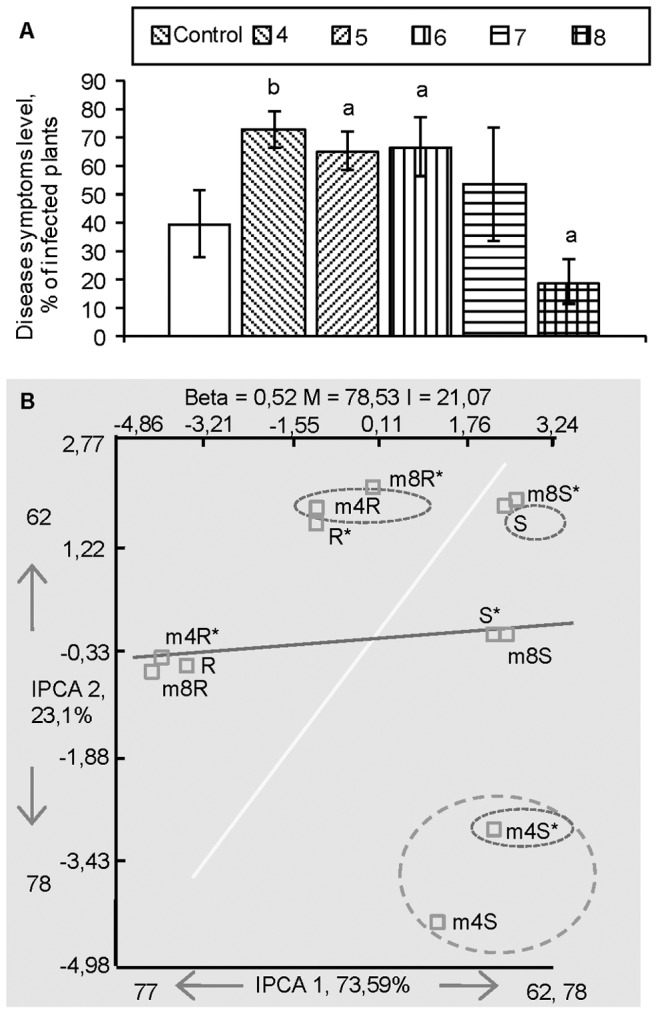
Disease resistance and terminal fragment length polymorphism (T-RFLP) analysis of endophyte communities in *Methylobacterium*-inoculated *in vitro* seedlings of Scots pine. (A) Resistance of *in vitro-*grown Scots pine to *Gremmeniella abietina* induced by *Methylobacterium extorquens* DSM13060 (m) applied at densities 10^4^, 10^5^, 10^6^, 10^7^ and 10^8^ CFU ml^−1^ (4, 5, 6, 7 and 8) and (B) analysis of the corresponding bacterial communities in shoots and roots at 10^4^ and 10^8^ densities. Disease resistance data are means ± SD (*n* = 5), letters indicate significant difference between treatments and control by Student’s *t*-test (a, b and c indicate P<0.05, 0.01 and 0.001, respectively). Cluster plots generated by Additive Main Effects and Multiplicative Interaction (AMMI) analysis are constructed from five T-RFLP replicates and contain the information on beta diversity (Beta), the percentage of the main (M) and interaction (I) effects, the principal T-RFs responsible for the data ordination for each of the interaction principal components axes (IPCA1 and 2), and the percentage of variance captured by each of the axes. Different shapes indicate grouping patterns.

Prior to T-RFLP analysis, the bacterial 720-bp 16S rDNA amplicons were cloned and sequenced to confirm the quality of PCR products, and bacterial sequences identical or similar with strains of *e.g. Bacillus pumilus*, *Pseudomonas fluorescens* and *Ralstonia taiwanensis* were identified. We attempted to identify the corresponding T-RFs, and four-bp offset was observed between the theoretical and experimental sizes of T-RFs. The main T-RFs responsible for data ordination could be matched with the following most closely related bacterial species: 62 bp (*P. fluorescens*), 74 bp (*Cupriacidius metallidurans*), 77 bp (*P. atrosepticum*) and 78 bp (*B. pumilus*). T-RFs corresponding to the *Methylobacterium* spp. inoculants were not among the principal components responsible for data ordination in any experiment. The average T-RF richness in all T-RFLP analyses varied from 4.4 to 7.0. In all T-RFLP profiles analyzed, there were at least two significant principal component axes (P<0.001) identified by F-test at 5% level. These first two axes captured the total variance from 88.0 to 96.99%. Beta diversity and the percentages of the main and interaction effects are shown in Fig. 1B, C, Fig. 2B and Fig. 3B.

In the *in vitro* experiment on *Methylobacterium* sp. IMBG290 inoculation of potato, tested against *P. atrosepticum*, the most important factor for treatment grouping was the pathogen infection, followed by the plant genotype and the *Methylobacterium* sp. IMBG290 inoculation (Fig. 1B). The challenge inoculation with *P. atrosepticum* induced significant changes in the bacterial communities. The difference between the treatments before versus after challenge inoculation was determined by the appearance of new T-RFs (72 bp, 286 bp, P<0.001 and 73 bp, P<0.01) along with the 77 bp (P<0.001) fragment originating from *P. atrosepticum* (Table S2). In addition, significant changes were observed for the majority of the T-RFs present, as the relative abundance of the 44-bp fragment (*Methylobacterium* sp. IMBG290) decreased (P<0.05) and the ratio of T-RFs 62 bp (*P. fluorescens*) and 78 bp (*B. pumilus*) increased (P<0.01 and P<0.001, respectively) after challenge inoculation. These changes were dependent on the potato genotype and on the pre-treatment with *Methylobacterium* sp. IMBG290. The shift in the community structure observed after *Methylobacterium* sp. IMBG290 treatment and challenge inoculation was associated with disease resistance: in plants showing resistance (cvs. Blue Congo and Pito) a 62-bp T-RF (*P. fluorescens*) was detected, while a 44 bp T-RF (*Methylobacterium* sp. IMBG290) disappeared (Table S2).

Because the induction of resistance by *Methylobacterium* sp. IMBG290 was dependent on the inoculation density, the innate endophyte community structures were studied in more detail in Blue Congo shoots and roots. Blue Congo plants were previously shown to consistently express enhanced disease resistance associated with the low inoculation density [17] and therefore were selected for the T-RFLP analysis. The analysis demonstrated that the root endophyte communities of all treatments grouped together. The shoot endophyte communities differed before and after the challenge inoculation in plants treated with the low and high inoculation densities of 10^5^ and 10^8^ CFU ml^−1^ (Fig. 1C). Two T-RFs (62 bp [*P*. *fluorescens*], P<0.01 and 78 bp [*B*. *pumilus*], P<0.05) were significantly different between these treatments (P<0.001 and P<0.01 before and after challenge inoculation, respectively). The shoot endophyte communities of plants treated with the high inoculation density were similar to that of the roots, and hosted a lower bacterial richness based on the number of T-RFs (Fig. 1C, Table S2). To assess the colonization by the *Methylobacterium* sp. IMBG290 endophyte using the two inoculation densities, the colonies growing from the plant tissue were counted after four weeks. When the low inoculation density was used, colonization was 3.45
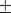
0.86 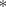
10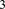 CFU g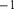 in the shoots and 1.74
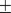
0.12 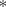
10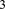 CFU g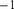 in the roots. At the high inoculation density, the colonization was 1
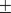
0.13 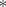
10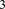 CFU g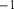 in the shoots and 0.3
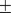
0.05 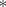
10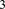 CFU g
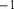
in the roots. The colonization using the two inoculation densities differed significantly in the roots (P<0.001) and shoots (P<0.01).

#### Greenhouse experiment

Since the endophyte communities of *in vitro*-grown plants are generally small, the changes in the community structures were further analyzed in greenhouse-grown plants. The cultivars Bellarosa and Yavir were inoculated with *Methylobacterium* sp. IMBG290 at two densities, 10^3^ and 10^5^ CFU mL^−1^. No significant effect on resistance towards *P. infestans* was observed (Fig. S1B), but resistance towards both bacterial pathogens *P. syringae* pv. tomato and *P. atrosepticum* was enhanced in Bellarosa plants regardless of the inoculation density (Fig. 2A, Fig. S1B). The experiment against *P. syringae* pv. tomato was selected for T-RFLP analysis of the innate endophyte communities. According to the analysis, the *Methylobacterium* sp. IMBG290 inoculation induced distinct changes in the bacterial community structure of shoots but not roots (Fig. 2C, Table S2), regardless of the fact that inoculations were done on the roots. Three T-RFs; 41 bp (F), 166 bp (F) (both at P<0.05) and 184 bp (F) (P<0.01) were significantly different in these treatments. The bacterial community of *Methylobacterium* sp. IMBG290-treated shoots grouped together with the root community, but after challenge inoculation these treatments segregated, T-RF 41 bp (R) (P<0.05) being significantly different between these treatments.

### Scots Pine (*Pinus sylvestris* L.)

To explore the community changes in response to endophyte inoculation and pathogen challenge in another plant species, we performed a similar *in vitro* experiment on Scots pine. When pine seedlings were inoculated with the endophyte *M. extorquens* DSM13060, resistance against *G. abietina* was observed at the high inoculation density (10^8^ CFU ml^−1^), whereas seedlings treated with low density (10^4^ CFU ml^−1^) became more susceptible to the pathogen (Fig. 3A). When the innate endophyte population structures were studied in these samples, treatment with *M. extorquens* DSM13060 significantly decreased the relative abundance of T-RF 44 bp (P<0.05) in the shoots (Fig. 3B, Table S2). Furthermore, the T-RF 62 bp (*P. fluorescens*) significantly decreased (P<0.05) when the low density of *M. extorquens* DSM13060 cells was used. After challenge inoculation, the T-RF of 221 bp disappeared (P<0.05) and the T-RF of 74 bp (*C. metallidurans*, P<0.05) disappeared in the root community from the samples treated with the low density of *M. extorquens* DSM13060 cells.

## Discussion

The present study was aimed to elucidate the changes occurring in innate endophytic communities associated with inoculation of beneficial endophytes, pathogens, and the resulting disease tolerance or susceptibility. The *Methylobacterium* spp. inoculant strains had a variable effect on plant disease resistance. These results can be explained largely by the fact that the tested host plant species, as well as pathogens used, differ from each other by defense mechanisms and disease strategies, respectively. Potato is an annual dicotyledonous crop plant that is propagated clonally, and relies largely on inducible defense responses [41]. Scots pine is a wind-pollinated coniferous tree having an efficient constitutive defense system, such as preformed resins and polyphenols [42], [43]. *Pectobacterium atrosepticum* is a necrotrophic pathogen, which elicits both salicylic acid (SA)- and JA-ET-dependent defense responses [44], whereas *Pseudomonas syringae* pv. tomato is a hemibiotroph that elicits the SA-defense signaling cascade and inhibits the JA(ET)-dependent defense responses [45], and *Gremmeniella abietina* is a fungal pathogen. Such large scale of plant-pathogen combinations were used to reveal whether endophyte inoculation induces a change in the innate endophyte community universally with respect to resistance or susceptibility to a pathogen.

Presence of a pathogen affects the innate endophyte community structure of a plant [8], [10], [23], [46], and in this paper, we show that endophyte inoculation can modulate the communities, to result in a divergent structure after pathogen challenge. Even though results of T-RFLP analysis are semi-quantitative due to PCR bias [47], statistically significant changes in the structures were observed in different combinations of plant species, pathogen, and *Methylobacterium* spp. inoculation. The endophyte community analysis indicated that the structures were dependent on *Methylobacterium* spp. inoculation density, plant genotype, and pathogen challenge. Most importantly, *Methylobacterium* spp.-inoculated plants challenged with the pathogen showed highly different endophyte communities compared to uninoculated control plants. Typically, the *Methylobacterium* spp. inoculation alone induced only small changes in the endophyte communities. However, the changes in the community structures induced by pathogen inoculation were different between controls and *Methylobacterium* spp.-treated plants, indicating that *Methylobacterium* spp. inoculation had modified the endophyte community responses towards pathogen challenge. Specifically, some of the appearing or disappearing T-RFs could be associated with resistance or susceptibility of the plant to the disease. For example, the relative abundance of *P. fluorescens* increased significantly after *Methylobacterium* sp. IMBG290 treatment and correlated with enhanced resistance towards *P. atrosepticum*. In the greenhouse experiment against *P. syringae* pv. tomato, three T-RFs were significantly different in endophytic bacterial communities of potato inoculated with *Methylobacterium* sp. IMBG290, coinciding with less leaf disease symptoms. When pine seedlings were inoculated with the endophyte *M. extorquens* DSM13060, one significantly different T-RF correlated with pathogen resistance.

The endophyte-induced changes occurred mainly in the shoot communities regardless of endophyte inoculation being done on the roots, indicating systemic effects. *Methylobacterium* sp. IMBG290 is an active colonizer of potato, residing in the leaves and stems of potato shoots [11], and *M. extorquens* DSM13060 has been isolated from pine shoot tips [48]. Neither of the *Methylobacterium* spp. endophyte strains used for plant inoculation have direct antagonistic activity towards the pathogens tested [49]. Microorganisms, irrespective of their mode of action, can modulate plant responses [16]–[18] and thereby affect innate endophyte communities. We have earlier analyzed the defense responses of *in vitro*-grown potato cultivar Blue Congo when inoculated at high and low inoculation densities with *Methylobacterium* sp. IMBG290 against *P. atrosepticum* [17]. In those studies the same results were obtained, low inoculation density resulting in resistance and high density leading to susceptibility towards the pathogen, but no obvious mechanism behind the phenomenon was identified. The antioxidant system of potato was moderately activated by the endophyte inoculation specifically at low inoculation density [17]. Modulation of the antioxidant system is important for plant defense [50], but it is not a direct defense mechanism, *i.e.* it does not kill or inhibit the growth of the pathogen. The infection by microbes induces production of reactive oxygen species, which in turn, activates the antioxidant system, including ascorbate and glutathione reducing enzymes [51]. Generally, the plant redox status is considered a candidate factor maintaining *status quo* in the plant-endophyte interaction (see *e.g.* [52]). Rocking this balance could affect the growth of individual, innate endophyte species inside the plant, seen as changes in the community structure by T-RFLP.

As endophyte communities were significantly different in resistant or susceptible plants in both potato and pine, there could be a (direct or indirect) link between the presence or absence of certain community members and disease tolerance. We have earlier seen that an inoculated bacterial strain can dramatically increase the numbers of specific endophytes inside the plant, even to a point of inducing outgrowth [5], [11], [26]. *In vitro*-grown plants typically host a far lower diversity of endophytes than field-grown plants, and the innate endophytic community is cultivar-specific, potentially including beneficial bacteria as well as latent pathogens [4]. Therefore, changes occurring in the plant due to external factors can affect growth of both beneficial and potentially harmful microorganisms, either positively or negatively [13], [21], [22]. If the populations of latent pathogens grow, the defense system of the plant host becomes compromised [53]–[55]. On the other hand, favorable conditions for growth of beneficial or protective endophytes due to endophyte inoculation can increase plant resistance. In potato cv. Blue Congo, sequence analysis indicated the presence of strains closely related to *P. fluorescens* and *Bacillus pumilus* that have earlier been reported as plant growth-promoting bacteria capable of triggering ISR [1], [3], [4].

Recently Doornbos *et al.* [56] reported that Arabidopsis mutants compromised in ISR differ from wild-type plants by their rhizosphere bacterial microflora, suggesting a potential link between the plant-associated bacterial community and the development of ISR. As endophytes have several ways of increasing plant resistance towards pathogens, the outcome of their concerted action defines the type of plant responses. Whether the endophyte-induced changes in the population structures of innate endophytic communities are responsible for plant resistance or susceptibility towards the pathogen, or simply a reaction to the plant responses, cannot be exclusively determined in this study. However, the study shows that changes in the endophyte communities correspond with plant responses and therefore the significance of the innate plant endophyte microbiome should be considered in future studies on plant defense.

## Supporting Information

Figure S1
**Effect of** ***Methylobacterium*** **inoculation on disease resistance of potato.** Resistance of (A) *in vitro-*grown potato cvs. Blue Congo, Timo, Pito, Matilda (B, T, P, M) to *Phytophthora infestans* and (B) greenhouse-grown potato cvs. Bellarosa (Br) and Yavir (Y*) towards *P. infestans* (Pi) and *Pectobacterium atrosepticum* (Pa). *Methylobacterium* sp. IMBG290 was applied at densities of 10^5^, 10^6^, 10^7^ and 10^8^ CFU ml^−1^ (5, 6, 7 and 8 respectively) to in vitro-grown plants (A) and at densities of 10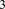 and 10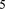 CFU mL
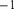
(3 and 5 respectively) to greenhouse-grown plants (B). Control – mock-treated plants. Data are means ± SD (*n* = 5). Letters indicate significant difference between the treatments and control by Student’s *t*-test (a, b and c indicate P<0.05, 0.01 and 0.001 respectively).(TIF)

Table S1 Summary of the experiments performed.
(DOC)

Table S2 Data matrices used for AMMI from three (Fig 1B, 1C, 3B) and five (Fig. 2B) T-RFLP replicates. Mb = T-RF corresponding to *Methylobacterium*, B = potato cv. Blue Congo, T = Timo, P = Pito, M = Matilda, m = *Methylobacterium* inoculation, number indicates inoculation density (log10 CFU ml^−1^), S = shoot, R = root, * = challenge inoculation.
(DOC)
