# Supplementary figures and images for: Methylobacterium-Induced Endophyte Community Changes Correspond with Protection of Plants against Pathogen Attack
Source: PLoS One. 2012 Oct 3;7(10):e46802. doi: 10.1371/journal.pone.0046802 (PMC3463518; doi:10.1371/journal.pone.0046802)

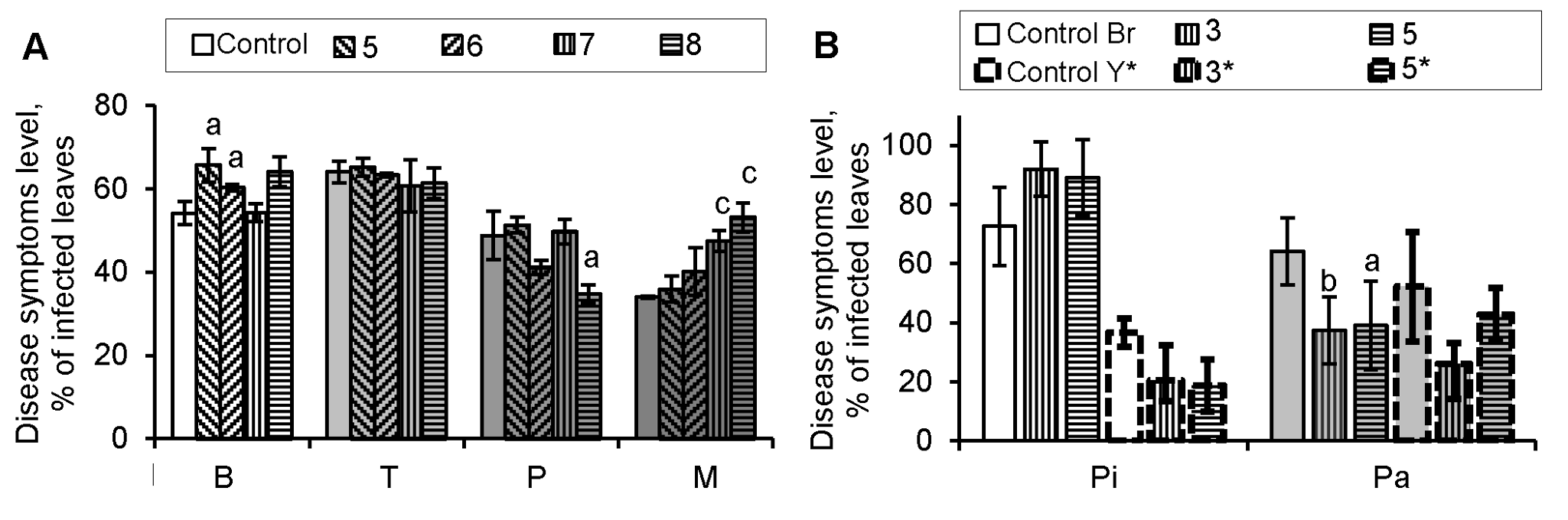

Supplement: Figure S1 — Effect of Methylobacterium inoculation on disease resistance of potato. Resistance of (A) in vitro-grown potato cvs. Blue Congo, Timo, Pito, Matilda (B, T, P, M) to Phytophthora infestans and (B) greenhouse-grown potato cvs. Bellarosa (Br) and Yavir (Y*) towards P. infestans (Pi) and Pectobacterium atrosepticum (Pa). Methylobacterium sp. IMBG290 was applied at densities of 105, 106, 107 and 108 CFU ml−1 (5, 6, 7 and 8 respectively) to in vitro-grown plants (A) and at densities of 10 and 10 CFU mL(3 and 5 respectively) to greenhouse-grown plants (B). Control – mock-treated plants. Data are means ± SD (n = 5). Letters indicate significant difference between the treatments and control by Student’s t-test (a, b and c indicate P<0.05, 0.01 and 0.001 respectively). (TIF) [file pone.0046802.s001.tif]
