# Supplementary material for: Methylobacterium-Induced Endophyte Community Changes Correspond with Protection of Plants against Pathogen Attack
Source: PLoS One. 2012 Oct 3;7(10):e46802. doi: 10.1371/journal.pone.0046802 (PMC3463518; doi:10.1371/journal.pone.0046802)
Supplement: Table S1 — Summary of the experiments performed. (DOC) [file pone.0046802.s002.doc]

**Table S1. Summary of the experiments performed**.

| **Experiments** | **Plant (cultivars)** | **Inoculation** | ***Methylobacterium* levels, CFU ml^-1^** | **Pathogen** | **T-RFLP** | **ARDRA and Sequence analysis** |
| --- | --- | --- | --- | --- | --- | --- |
| Experiment 1  (*in vitro*) | Potato (Timo, Pito, Matilda, Blue Congo) | *Methylobacterium* sp. strain IMBG290 | 10^5^, 10^6^, 10^7^, 10^8^ | *^a^Pa* | Timo, Pito, Matilda: 10^5^ CFU ml-1; shoots  Blue Congo: 10^5^, 10^8^ CFU ml^-1^; shoots, roots | 120 clones |
|  |  |  | 10^5^, 10^6^, 10^7^, 10^8^ | *^a^Pi* |  |  |
|  | Pine | *Methylobacterium* *extorquens* strain DSM13060 | 10^4^, 10^5^, 10^6^, 10^7^, 10^8^ | *^a^Ga* | 10^4^, 10^8^ CFU ml^-1^; shoots, roots | 24 clones |
| Experiment 2  (greenhouse) | Potato (Bellarosa, Yavir) | *Methylobacterium* sp. strain IMBG290 | 10^3^, 10^5^ | *^a^Pa* |  |  |
|  |  |  | 10^3^, 10^5^ CFU ml^-1^ | *^a^Pi* |  |  |
|  |  |  | 10^3^, 10^5^ CFU ml^-1^ | *^a^Pst* | Bellarosa: 10^3^, 10^5^ CFU ml^-1^; shoots |  |

^a^*Pa*, *Pi*, *Ga*, *Pst* – pathogens *Pectobacterium atrosepticum*, *Phytophthora infestans*, *Gremmeniella abietina*, *Pseudomonas syringae* pv. *tomato* DC3000 respectively used for challenge inoculation.
