# Supplementary material for: Methylobacterium-Induced Endophyte Community Changes Correspond with Protection of Plants against Pathogen Attack
Source: PLoS One. 2012 Oct 3;7(10):e46802. doi: 10.1371/journal.pone.0046802 (PMC3463518; doi:10.1371/journal.pone.0046802)
Supplement: Table S2 — Data matrices used for AMMI from three (Fig 1B, 1C, 3B) and five (Fig. 2B) T-RFLP replicates. Mb = T-RF corresponding to Methylobacterium, B = potato cv. Blue Congo, T = Timo, P = Pito, M = Matilda, m = Methylobacterium inoculation, number indicates inoculation density (log10 CFU ml−1), S = shoot, R = root, * = challenge inoculation. (DOC) [file pone.0046802.s003.doc]

**Table S2.** Data matrices used for AMMI from three (Fig 1B, 1C, 3B) and five (Fig. 2B) T-RFLP replicates. Mb= T-RF corresponding to *Methylobacterium*, B=potato cv. Blue Congo, T=Timo, P=Pito, M=Matilda, m= *Methylobacterium* inoculation, number indicates inoculation density (log10 CFU ml^-1^), S= shoot, R=root, *=challenge inoculation.

Fig. 1B

| Treatment/T-RF | 44 (Mb) | 62 | 71 | 72 | 73 | 74 | 75 | 77 | 78 | 286 |
| --- | --- | --- | --- | --- | --- | --- | --- | --- | --- | --- |
| B | 0 | 36,95 | 0 | 0 | 0 | 22,51 | 0 | 0 | 40,54 | 0 |
| T | 0 | 21,85 | 0 | 0 | 0 | 36,35 | 0 | 0 | 41,8 | 0 |
| P | 0 | 34 | 0 | 0 | 0 | 29,12 | 0 | 0 | 36,87 | 0 |
| M | 0 | 23,65 | 0 | 0 | 0 | 27,7 | 0 | 0 | 48,64 | 0 |
| mB | 0 | 0 | 0 | 0 | 0 | 10,82 | 7,94 | 0 | 81,24 | 0 |
| mT | 8,43 | 23,22 | 0 | 0 | 0 | 21,21 | 0 | 0 | 47,14 | 0 |
| mP | 9,47 | 19,96 | 1,34 | 0 | 0 | 28,91 | 1,79 | 0 | 38,53 | 0 |
| mM | 23,6 | 22,84 | 0,89 | 0 | 0 | 26,51 | 1,09 | 0 | 25,07 | 0 |
| B* | 0 | 27,34 | 0 | 39,33 | 4,21 | 0 | 7,81 | 5,91 | 9,15 | 6,24 |
| T* | 0 | 0 | 0 | 34,17 | 11,08 | 0 | 0 | 49,47 | 0 | 5,29 |
| P* | 0 | 0 | 0 | 23,46 | 0 | 16,51 | 0 | 55,12 | 0 | 4,91 |
| M* | 0 | 0 | 0 | 5,56 | 1,51 | 0 | 0 | 92,93 | 0 | 0 |
| mB* | 0 | 24,81 | 0 | 61,22 | 3,71 | 0 | 0 | 1,68 | 0 | 8,58 |
| mT* | 0,78 | 0 | 0,58 | 36,77 | 14,64 | 0 | 0 | 42,43 | 0 | 4,79 |
| mP* | 0 | 4,99 | 3,02 | 1,36 | 0 | 9,56 | 0 | 79,47 | 0 | 1,6 |
| mM* | 2,2 | 0 | 0 | 22,72 | 4,04 | 0 | 0 | 68,05 | 0 | 2,99 |

Fig 1C

| Treatment/T-RF | 44 (Mb) | 62 | 72 | 73 | 74 | 75 | 77 | 78 | 88 | 286 |
| --- | --- | --- | --- | --- | --- | --- | --- | --- | --- | --- |
| S | 0 | 10,07 | 0 | 0 | 29,97 | 4,2 | 0 | 55,76 | 0 | 0 |
| m5S | 0 | 0 | 0 | 0 | 7,99 | 9,55 | 0 | 82,45 | 0 | 0 |
| m8S | 0,88 | 43,95 | 0 | 0 | 5,7 | 5,46 | 0,54 | 43,48 | 0 | 0 |
| R | 0 | 72,64 | 0 | 0 | 5,48 | 10,4 | 0,6 | 10,86 | 0 | 0 |
| m5R | 8,3 | 70,44 | 0 | 0 | 2,22 | 4,84 | 0 | 14,19 | 0 | 0 |
| m8R | 0,57 | 80,3 | 0 | 0 | 4,33 | 8,23 | 2,33 | 4,24 | 0 | 0 |
| S* | 0 | 27,09 | 38,98 | 4,19 | 0 | 7,71 | 5,84 | 9,06 | 0,92 | 6,21 |
| m5S* | 0 | 23,56 | 49,87 | 1,58 | 0 | 0,56 | 9,44 | 6,41 | 1,95 | 6,64 |
| m8S* | 0 | 60,61 | 14,14 | 2,07 | 0 | 2,67 | 20,5 | 0 | 0 | 0 |
| R* | 0 | 82,26 | 0 | 0 | 4,16 | 4,02 | 9,56 | 0 | 0 | 0 |
| m5R* | 0 | 64,93 | 0 | 0 | 8,11 | 9,36 | 17,6 | 0 | 0 | 0 |
| m8R* | 10,9 | 63,08 | 0 | 0 | 1,71 | 6,85 | 17,46 | 0 | 0 | 0 |

Fig 2B

| T-RF/Treatment | S | mS | R | mR | S* | mS* |
| --- | --- | --- | --- | --- | --- | --- |
| 41F | 40,4 | 0 | 0 | 0 | 0 | 0 |
| 44F | 0 | 0 | 0 | 0 | 5,44 | 0 |
| 51F | 0 | 0 | 0 | 0 | 3,79 | 0 |
| 166F | 0,42 | 2 | 0 | 0 | 1,24 | 20,48 |
| 173F | 0 | 1,7 | 0 | 0 | 6,78 | 3,28 |
| 178F | 0 | 0 | 0 | 0 | 0 | 0,54 |
| 184F | 55,28 | 91,33 | 89,65 | 100 | 39,07 | 53,42 |
| 185F | 0 | 0 | 0 | 0 | 40,59 | 20,11 |
| 186F | 0,56 | 0 | 0 | 0 | 1,4 | 0,6 |
| 350F | 1,39 | 1,7 | 10,35 | 0 | 1,45 | 1,57 |
| 408F | 1,95 | 3,27 | 0 | 0 | 0,24 | 0 |
| 40R | 0 | 0 | 0 | 0 | 27,83 | 7,16 |
| 41R | 91,21 | 100 | 91,71 | 100 | 0 | 83,67 |
| 185R | 8,79 | 0 | 0 | 0 | 72,17 | 9,17 |
| 396R | 0 | 0 | 2,95 | 0 | 0 | 0 |
| 400R | 0 | 0 | 5,34 | 0 | 0 | 0 |

Fig 3B

| Treatment/T-RF | 44 | 62 | 71 | 74 | 75 | 77 | 78 | 221 |
| --- | --- | --- | --- | --- | --- | --- | --- | --- |
| S | 1,38 | 74,68 | 0 | 7,13 | 6,45 | 0 | 9,33 | 1,04 |
| m4S | 0 | 42,46 | 0,99 | 6,08 | 3,38 | 10,72 | 35,16 | 1,21 |
| m8S | 0 | 65,57 | 0,86 | 7,32 | 6,95 | 0 | 18,31 | 0,99 |
| R | 1,6 | 45,12 | 0 | 2,91 | 2,44 | 46,41 | 0 | 1,51 |
| m4R | 0,71 | 65,44 | 0 | 2,41 | 2,27 | 28,46 | 0 | 0,71 |
| m8R | 1,46 | 43,65 | 0 | 0,89 | 1,72 | 52,29 | 0 | 0 |
| S* | 0 | 61,57 | 2,5 | 14,72 | 9,35 | 0 | 11,86 | 0 |
| m4S* | 0 | 47,4 | 4,15 | 10,36 | 12,78 | 0 | 25,32 | 0 |
| m8S* | 0 | 78,24 | 0 | 0 | 8,24 | 0 | 13,53 | 0 |
| R* | 1,07 | 63,57 | 0 | 2,31 | 4,81 | 28,23 | 0 | 0 |
| m4R* | 3 | 45,46 | 0 | 0 | 1,31 | 50,23 | 0 | 0 |
| m8R* | 0,62 | 68,94 | 0 | 6,45 | 4,91 | 19,07 | 0 | 0 |
